# Eco-Friendly Hydrogel Beads from Seashell Waste for Efficient Removal of Heavy Metals from Water

**DOI:** 10.3390/polym16233257

**Published:** 2024-11-23

**Authors:** Zaineb Mchich, Daniela Simina Stefan, Rachid Mamouni, Nabil Saffaj, Magdalena Bosomoiu

**Affiliations:** 1 Team of Biotechnology, Materials, and Environment, Faculty of Sciences, Ibn Zohr University, Agadir BP 8106, Morocco; zaineb.mchich@edu.uiz.ac.ma (Z.M.); n.saffaj@uiz.ac.ma (N.S.); 2 Department of Analytical Chemistry and Environmental Engineering, Faculty of Chemical Engineering and Biotechnologies, National University of Science and Technology Politehnica of Bucharest, 1-7 Polizu Street, 011061 Bucharest, Romania; magdalena.bosomoiu@upb.ro

**Keywords:** eco-friendly polymers, *Cellana Tramoscrica* seashells, hydrogel beads, heavy metals

## Abstract

The objective of this study is to develop a calcium carbonate-based adsorbent derived from *Cellana Tramoscrica* seashells, incorporated into a sodium alginate matrix (Na-Alg@CTs) to form hydrogel beads, for the efficient removal of Cu (II) and Zn (II) heavy metals from aqueous solutions. XRD, SEM/EDS, and FTIR analysis confirm the successful synthesis and characterization of the fabricated adsorbent. The adsorption study of Cu (II) and Zn (II) onto Na-Alg@CTs hydrogel beads revealed that the Langmuir model was the most suitable for characterizing the adsorption isotherms, suggesting monolayer coverage. Na-Alg@CTs exhibited a maximum Langmuir adsorption capacity of 368.58 mg/g and 1075.67 mg/g for Cu (II) and Zn (II), respectively. Additionally, the kinetics followed the pseudo-second-order model, indicating that the adsorption process is primarily governed by chemisorption. The thermodynamic study suggests that the uptake of metal ions on Na-Alg@CTs hydrogel beads is spontaneous and endothermic. The exceptional adsorption capacity, eco-friendly nature, and low-cost characteristics of Na-Alg@CTs hydrogel beads make them an ideal adsorbent for the removal of Cu (II) and Zn (II) from wastewater.

## 1. Introduction

In recent decades, the rapid expansion of industrialization and urbanization has resulted in the release of industrial effluents, exhaust gases, and solid waste into the environment, both directly and indirectly, leading to significant water, soil, and atmosphere pollution [1,2]. Of particular concern are heavy metals, which pose serious risks to human health [3]. These metals, characterized by their significant contributions to water pollution, include elements such as copper, zinc, mercury, cadmium, chromium, lead, manganese, and nickel [4]. The primary sources of heavy metal pollution are industrial activities such as smelting, electroplating, electrolysis, and mining, as well as using chemicals in paints, pharmaceuticals, and pesticides. The wastewater generated by these industries is often laden with heavy metal ions, leading to their accumulation in natural water bodies and causing severe environmental contamination [5,6].

Among various metals, copper and zinc are essential yet potentially toxic when present in high concentrations. Copper is vital for several biological functions, including the growth and development of the body and the maturation of the nervous, hematopoietic, and skeletal systems [7,8]. However, excessive copper exposure often from contaminated water due to copper pipes, agricultural pesticides, and industrial discharges can be hazardous. Excessive copper accumulation can lead to digestive disorders, liver and kidney damage, and neurological imbalances [9,10,11]. Similarly, zinc is crucial for protein synthesis, immune function, and wound healing [12]. Nevertheless, overexposure to zinc, commonly from water contaminated by galvanized materials, fertilizers, and certain industrial products, can cause gastrointestinal issues and disrupt the immune system [13]. Elevated concentrations of these metals in aquatic ecosystems can be toxic to organisms, impairing their growth and reproduction. Therefore, managing copper and zinc pollution is a priority for health, scientific, and environmental authorities. These bodies are focusing on effective detoxification methods to safeguard human health and the environment [14]. Among the various remediation techniques developed are catalytic ozonation [15], ion exchange [16], coagulation-flocculation [17], photocatalysis [18,19], membrane filtration [20], and adsorption [21]. Of these, adsorption is particularly favored due to its simplicity, ease of application, use of low-cost materials, capacity to treat large volumes of wastewater, and recyclability [22,23]. The development of adsorbents using biomass waste as a substitute for activated carbon enhances the cost-effectiveness of the adsorption process.

Aquaculture of shellfish (such as squid, oysters, abalones, and mussels) is developing rapidly, leading to an increase in waste produced from shellfish [24]. Shell powder is commonly a source of calcium carbonate (CaCO_3_) and is known as a natural adsorbent for heavy metals due to its high specific surface area, high porosity, and low cost [25]. To address the limitations associated with the powdered form, considerable efforts have been directed toward developing low-cost composites, such as membranes, fibers, and beads [26]. In this regard, the utilization of polysaccharide adsorbent materials for the fabrication of mouldable composites has gained increasing prominence, offering a promising strategy to overcome these challenges [27]. Sodium alginate (Na-Alg), a natural water-soluble salt derived from brown seaweed, has emerged as a key material due to its high bioavailability and straightforward extraction process [28]. As a linear polysaccharide, sodium alginate consists of α-L-guluronic acid (G) and β-D-mannuronic acid (M) residues [29]. Na-Alg possesses a distinctive property that enables it to form hydrogels by substituting sodium ions in guluronic acid residues with divalent cations, including calcium (Ca^2+^), barium (Ba^2+^), strontium (Sr^2+^), … [30]. This ion exchange process results in the formation of a three-dimensional network. Consequently, the utilization of Na-Alg to encapsulate CaCO_3_ generated from seashells represents a sophisticated methodology that is designed to minimize mass loss, reduce regeneration costs, and enhance the efficiency of large-scale water treatment.

This research paper aims to investigate the conversion of CaCO₃ generated from *Cellana Tramoserica* shells (CTs) into valuable green biocomposites by incorporating it into the Na-Alg matrix, which is renowned for its excellent adsorption properties for heavy metals. The biocomposite physicochemical properties were analyzed using scanning electron microscopy, X-ray diffraction, energy-dispersive X-ray spectrometer, and Fourier-transformed infrared spectroscopy. Batch adsorption experiments were conducted to assess the adsorption performance of Na-Alg@CTs hydrogel beads for the removal of Cu (II) and Zn (II) ions. These experiments systematically evaluated the hydrogel beads under a variety of conditions, including different adsorbent dosages, pH levels, initial ion concentrations, temperatures, and contact time.

## 2. Materials and Methods

### 2.1. Materials

The chemicals utilized in the research were Na-Alg, CuSO_4_·5H_2_O, Zn (NO_3_)_2_·6H_2_O, HCl, NaOH, CaCl_2_ (manufacturer of all p.a. Fluka), all reagents were of analytical grade. The *Cellana Tramoscrica* seashells were procured from Cap Ghir beach in Agadir, Morocco.

### 2.2. Methods

The preparation of *Cellana Tramoscrica* seashells was conducted under the protocol outlined by [31]. The shells were first subjected to an extensive cleaning process using distilled water (DW) to remove any remaining sand particles and then subjected to treatment with 0.1 M of HCl for one night to remove organic substances. Following this, the shells were washed again and dried at 120 °C. Finally, the shells were crushed and sieved to 80 µm. The resulting powder was then washed and dried at 70 °C, after which it was labeled as CTs.

The synthesis process for CTs encapsulation was initiated with the dissolution of 1 g of Na-Alg in 100 mL of DW, which was then stirred thoroughly on a magnetic stirrer for 3 h until complete dissolution and the disappearance of air bubbles. Thereafter, 1 g of CTs was added to the alginate gel. To obtain a homogeneous mixture, the mixing process was continued overnight. Subsequently, the solution was added dropwise to a 1% (*w*/*v*) calcium chloride solution via a syringe, forming roughly uniform-sized beads. The hydrogel beads were subsequently washed with DW to eliminate any residual calcium chloride, stored in a bottle containing DW, and labeled as Na-Alg@CTs hydrogel beads. Figure S1 shows the detailed process of hydrogel beads preparation.

### 2.3. Characterization of Na-Alg@CTs Hydrogel Beads

The hydrogel beads of the Na-Alg@CTs hydrogel biocomposite were characterized through a series of techniques, including X-ray diffraction using a Bruker CCD-Apex instrument in the 2θ range of 6° to 60° and scanning electron microscopy (SEM) using a Quanta Inspect F50, FEI Company, Eindhoven, The Netherlands. Furthermore, the samples were analyzed using an energy dispersive X-ray spectrometer (EDS) with MnK resolution of 133 eV, Fourier transformed infrared spectroscopy (FT-IR) using a Nicolet iS50FT-IR (Nicolet, Pittsfield, MA, USA) spectrometer equipped with a DTGS detector which provides information with a high sensitivity in the range of 4000 cm^−1^ and 400 cm^−1^ at a resolution of 4 cm^−1^.

The point of zero charge (pH _PZC_) of Na-Alg@CTs hydrogel beads was determined following the established protocol outlined in previous studies [32,33].

### 2.4. Adsorption Experiments

The metal ions Cu (II) and Zn (II) adsorption experiments onto Na-Alg@CTs hydrogel beads were carried out in a batch mode. To ascertain the impact of multiple variables, 0.25 g/L of hydrogel beads was introduced into 50 mL of Cu (II) or Zn (II) solutions. Multiple parameters were examined, including pH (3–6), time (5–180 min), dose (0.25–1.75 g/L), and initial concentration (5–200 mg/L) for Cu (II) and (5–500 mg/L) for Zn (II). Subsequently, the beads were removed from the solution after the specified time interval, and the residual Cu (II) and Zn (II) concentrations were quantified by flame absorption spectroscopy (Analytik Jena ContrAA 300, Bucharest, Romania). The removal percentage Equation (1) and adsorption capacity Equation (2) of Cu (II) and Zn (II) on Na-Alg@CTs hydrogel beads were calculated using the following equations.

(1) $$\%\;R=\frac{C_{i}-C_{e}}{C_{i}}\times 100$$

(2) $$Q_{e}=\frac{C_{i}-C_{e}}{m}\times V_{s}$$

where C_i_ (ppm) is the initial concentration, C_e_ (ppm) is the equilibrium concentration, m (mg) is the bio-adsorbent dose, and Vs (mL) is the solution volume.

## 3. Results and Discussion

### 3.1. FT-IR Analysis of Na- Alg@CTs Hydrogel Beads

**Figure 1:**
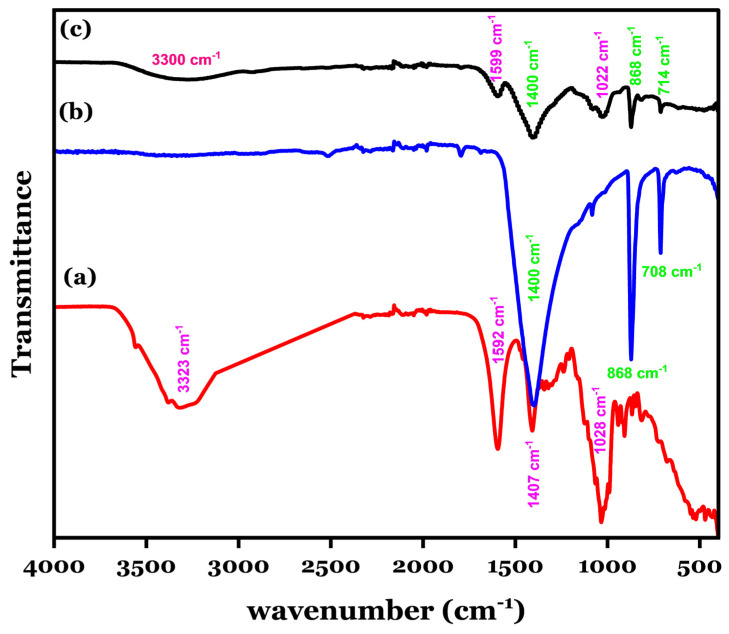
FT-IR analysis for (**a**) Na-Alg, (**b**) CTs, and (**c**) Na-Alg@CTs hydrogel beads.

The functional groups in Na-Alg (a), CTs (b), and Na-Alg@CTs hydrogel beads (c) were analyzed using FT-IR, as shown in Figure 1. The spectrum of Na-Alg exhibits four distinct bands corresponding to specific functional groups. The broad band at 3223 cm^−1^ is attributed to hydroxyl groups (-OH), while the bands at 1407 cm^−1^ and 1592 cm^−1^ are associated with symmetric and asymmetric stretching vibrations of carboxylate groups (-COO^−^), respectively. The band at 1028 cm^−1^ is linked to the -C-O-H stretching of alcoholic groups [34]. In the CTs spectrum, the bands at 1400 cm^−1^, 708 cm^−1^, and 868 cm^−1^ correspond to carbonate groups (CO₃^2−^) [35,36]. After encapsulation and the formation of Na-Alg@CTs hydrogel beads, the spectrum shows a reduction in the intensities of the characteristic bands from both CTs and Na-Alg, along with slight shifts in band positions. These changes suggest successful interaction and complexation between the components [37].

### 3.2. X-Ray Diffraction of Na-Alg@CTs Hydrogel Beads

**Figure 2:**
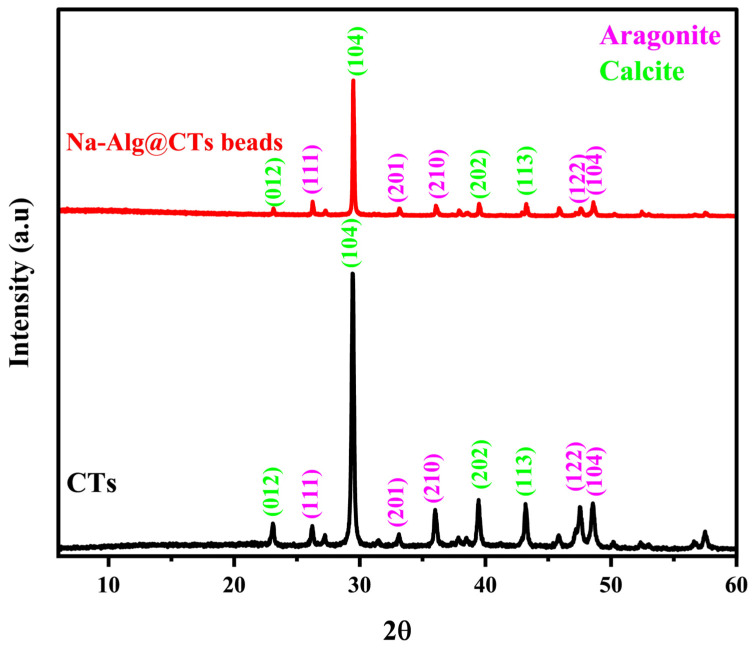
XRD of CTs and Na-Alg@CTs hydrogel beads.

The crystallography and phase characteristics of CTs and Na-Alg@CTs hydrogel beads were determined through XRD. As shown in Figure 2, the CTs exhibit a biphasic nature [38], with a dominant calcite phase characterized by peaks at 2θ values of 23.11° (012), 29.56° (104), 39.47° (113), and 43.23° (202). The secondary aragonite phase is identified by peaks at 2θ values of 26.23° (111), 33.13° (201), 36.01° (210), 45.87° (122), and 48.57° (104). The diffractograms of Na-Alg@CTs biocomposite confirm the successful dispersion of CTs within the Na-Alg matrix, as indicated by the reduced intensity of the peaks associated with CTs [39]. The absence of additional peaks indicates that the beads are pure, confirming that the crystallinity and purity of the CTs remain unaffected after successful encapsulation within Na-Alg beads [40].

### 3.3. SEM/EDS Analysis of Na-Alg@CTs Hydrogel Beads

**Figure 3:**
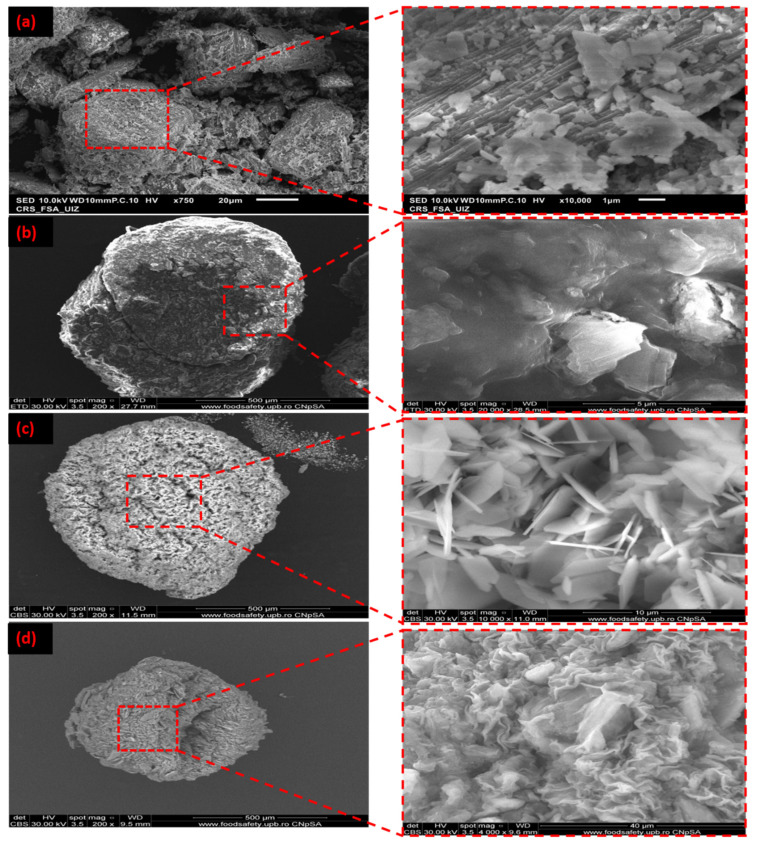
SEM of (**a**) CTs, (**b**) Na-Alg@CTs, (**c**) Cu (II)-Na-Alg@CTs, and (**d**) Zn (II)-Na-Alg@CTs.

The morphologies of the CTs and Na-Alg@CTs beads are illustrated in Figure 3, both in their original state and following Cu (II) and Zn (II) ions adsorption. The micrograph of the CTs (Figure 3a) reveals a lamellar structure with an irregular distribution of agglomerations on the surface [41]. As illustrated in Figure 3b, the Na-Alg@CTs beads exhibit a spherical morphology with an irregular and relatively rough surface [42]. The CTs are well dispersed in the matrix, as shown by SEM micrographs of the bio-nanocomposite beads. After the adsorption of two metal ions, changes in the surface are observed as a result of the uptake of Cu (II) (Figure 3c) and Zn (II) (Figure 3d) by the surface of Na-Alg@CTs hydrogel beads.

The EDS spectrum, along with the atomic percentages of the constituent elements in CTs and Na-Alg@CTs hydrogel beads before and after the adsorption of Cu (II) and Zn (II) are presented in Figure S2. The EDS spectrum of CTs (Figure S2a) indicates that the primary elements are oxygen (O = 66.35%), calcium (Ca = 16.92%), and carbon (C = 16.73%). After the encapsulation process (Figure S2b), these elements (Ca, C, and O) remain, while sodium (Na = 15.52%) from Na-Alg is also detected. Following the adsorption of Cu (II) and Zn (II) by the Na-Alg@CTs hydrogel beads, new peaks corresponding to Cu and Zn elements appear, as illustrated in Figure S2c and Figure S2d, respectively, confirming the uptake of metal ions on the surface of the biocomposite.

### 3.4. Adsorption Study of Cu (II) and Zn (II) onto Na-Alg@CTs Hydrogel Beads

#### 3.4.1. Na-Alg@CTs Hydrogel Beads Dose Effect

**Figure 4:**
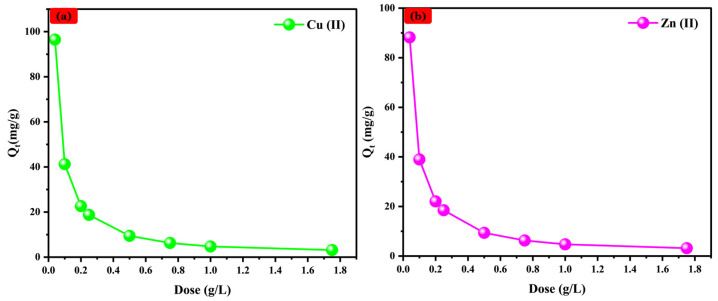
Na-Alg@CTs beads dose effect on the adsorption of (**a**) Cu (II) and (**b**) Zn (II).

A series of Na-Alg@CTs hydrogel beads ratios (m/V) was mixed with 50 mL of Cu (II) and Zn (II) solutions, each having an initial concentration of 5 ppm and agitated for 180 min. Figure 4 illustrates the relationship between the adsorbent dose and the adsorbed quantity of Cu^2+^ and Zn^2+^ cations. As illustrated, the adsorption capacity exhibited a declining trend. This observation can be attributed to the presence of higher amounts of Na-Alg@CTs beads, which can lead to agglomeration, causing overlapping of adsorption sites and a reduction in the effective surface area of the adsorbents. Equilibrium is attained at a mass of 0.25 g/L. Consequently, the optimal dose of the adsorbent was identified as 0.25 g/L for subsequent study.

#### 3.4.2. pH Effect

**Figure 5:**
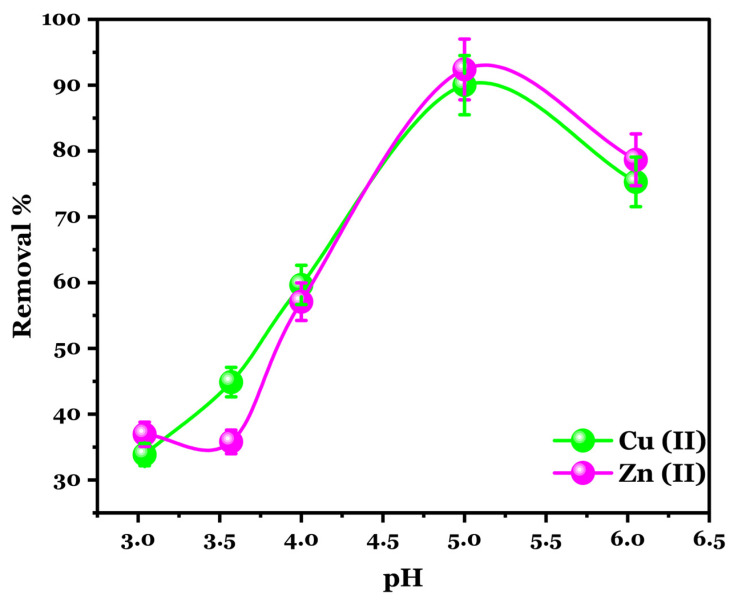
Effect of pH on the adsorption of Cu (II) and Zn (II) onto Na-Alg@CTs beads.

It is essential to investigate the effect of pH on the adsorption of Cu (II) and Zn (II) onto Na-Alg@CTs hydrogel beads, as pH is a key factor influencing pollutant availability, the surface charge of the adsorbent, and the interactions between the pollutants and the adsorbent [43]. Since Cu (II) and Zn (II) tend to precipitate at pH levels above 6, and Na-Alg@CTs beads shrink in solutions with a pH of 2, the pH range of 3–6 was selected to investigate its impact on the adsorption of both metals’ ions [44]. As shown in Figure 5, the removal rate of Cu (II) and Zn (II) ions increases significantly from 33.65% and 37.03% to 90.15% and 92.69% as the pH rises from 3 to 5, followed by a decrease at pH 6. The highest adsorption is achieved at a pH of 5. The relatively low adsorption capacity of Cu (II) and Zn (II) at pH 3 refers to the higher concentration of H⁺ ions in the solution competing with metal ions for active sites [45].

#### 3.4.3. Adsorption Isotherm

**Figure 6:**
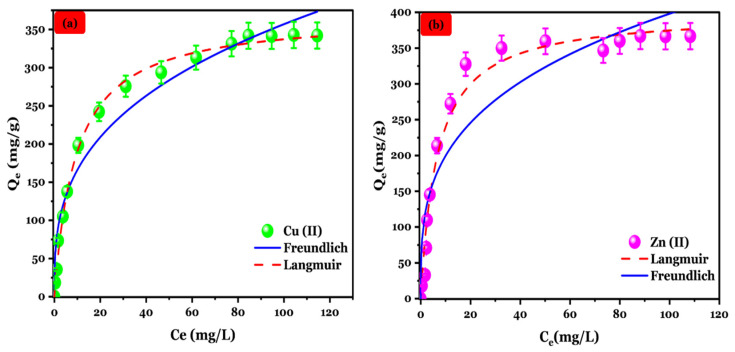
Isotherm fitting for (**a**) Cu (II) and (**b**) Zn (II) adsorption on Na-Alg@CTs hydrogel beads.

The study of adsorption isotherms is essential for understanding the adsorption behavior of a system and assessing its overall feasibility [46]. As illustrated in Figure 6, the adsorbed quantity of Cu (II) and Zn (II) metal ions onto Na-Alg@CTs hydrogel beads demonstrated an increase with the initial concentration increment. This phenomenon is attributed to the dynamic force increment that overcomes the resistance to mass transfer of metal ions from the solution to the Na-Alg@CTs hydrogel beads’ surface. The experimental data were evaluated using the Freundlich Equation (1) and Langmuir Equation (2) isotherm models. Their corresponding nonlinear equations are described below [47,48].

(3) $$Q_{e}=K_{e}C_{e}^{\frac{1}{n}}$$

(4) $$Q_{e}=\frac{Q_{m}K_{L}C_{e}}{1+K_{L}C_{e}}$$

where Q_e_ (mg/g), Q_m_ (mg/g), and K_L_ represent the amount of dye adsorbed per unit mass of adsorbent, the maximum adsorption capacity, and the Langmuir constant (in L/mg), respectively. K_F_ and n are the Freundlich constants associated with adsorption capacity and intensity, respectively.

**Figure 7:**
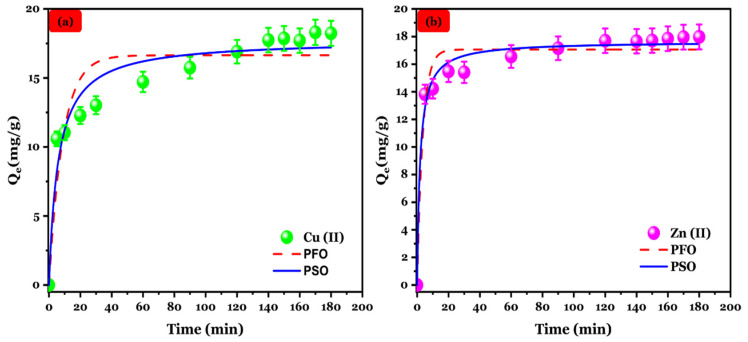
Kinetic fitting of (**a**) Cu (II) and (**b**) Zn (II) adsorption onto Na-Alg@CTs beads.

Figure 7 depicts the fitting curve of the experimental data, and the corresponding parameters are summarized in Table 1. Given the higher correlation coefficient, lower root mean square error (RMSE), and lower χ^2^, the Langmuir model is deemed to be an appropriate fit for the adsorption of Zn (II) and Cu (II) onto Na-Alg@CTs hydrogel beads suggesting that adsorption occurs in a monolayer on a surface with uniformly distributed active sites. Additionally, it assumes that the heat of adsorption remains constant, regardless of the extent of surface coverage, implying that there is no interaction between adsorbed molecules [49]. Moreover, the Langmuir model yielded an estimated maximum adsorption capacity of 368.875 mg/g and 1075.676 mg/g for Cu (II) and Zn (II), respectively, which closely aligned with the experimentally measured value of 342.09 mg/g and 1032.371 mg/g. These results are consistent with previous research on the adsorption of Zn (II) and Cu (II) [50].

#### 3.4.4. Adsorption Kinetic

The influence of contact time on the removal of Cu (II) and Zn (II) onto the Na-Alg@CTs hydrogel beads biocomposite was conducted, as illustrated in Figure 7. Cu (II) and Zn (II) adsorption capacity onto the Na-Alg@CTs exhibited a rapid increase during the initial 20 min, followed by a gradual deceleration, until reaching equilibrium. The initial rapid adsorption can be attributed to active site availability on Na-Alg@CTs hydrogel beads. However, as the adsorption time progressed, the available active sites became progressively occupied with a decrease in concentration gradient, leading to a diminished adsorption rate, and the system eventually approached an equilibrium state [51].

To elucidate the mechanisms of Cu (II) and Zn (II) adsorption on the Na-Alg@CTs hydrogel beads, the adsorption kinetic data were analyzed using pseudo-first-order (PFO), pseudo-second-order (PSO), and intraparticle diffusion kinetic (IPD) models. The non-linear equations corresponding to applied kinetic models are presented below [49,50,51]:

(5) $$Q_{t}=Q_{1}(1-\exp-K_{1}t)$$

(6) $$Q_{t}=\frac{K_{2}Q_{2}t}{1+K_{2}Q_{2}t}$$

(7) $$Q_{t}=K_{IPD}t^{\frac{1}{2}}+C$$

where the variables Q_t_, K_1_, and K_2_ correspond to the adsorbed quantity at a given instant (min), the constant rate of the PFO, and the constant rate of the PSO, respectively. K_IPD_ and C are constant rate of IPD and boundary layer thickness, respectively.

As summarized in Table 2 and based on the higher correlation coefficient (R^2^) and lower χ^2^ values, the PSO model provides a better fit to describe the adsorption kinetic of Cu (II) and Zn (II) than the PFO model. Additionally, the calculated Qe (mg/g) value predicted by the PSO for both metal ions is closer to the experimental value. Consequently, the Cu (II) and Zn (II) adsorption process on Na-Alg@CTs hydrogel beads follows the PSO kinetic model suggesting that the adsorption process is primarily governed by a chemisorption mechanism [52]. Furthermore, the higher rate constant (k) observed for Zn (II) further supports the conclusion that Zn (II) exhibits faster adsorption kinetics compared to Cu (II) [53].

**Figure 8:**
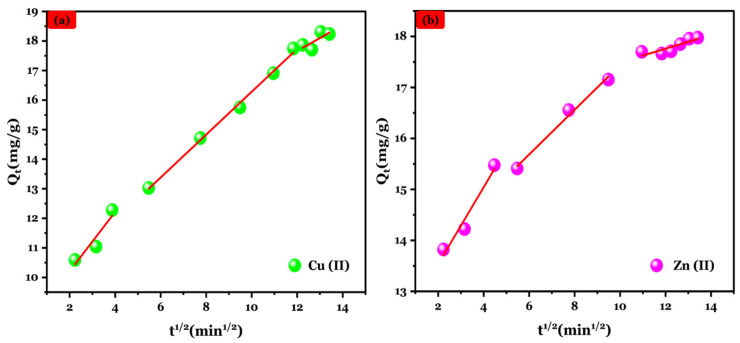
IPD model plot of (**a**) Cu (II) and (**b**) Zn (II) adsorption by Alg@CTs hydrogel beads.

The IPD model was applied to get a deeper insight into the mass diffusion mechanism at the liquid–solid interface. Figure 8 illustrates that the IPD model plots correspond to Cu (II) and Zn (II) adsorption by Na-Alg@CTs revealing three distinct phases. The first phase corresponds to the rapid transfer of Cu (II) and Zn (II) ions from solution to Na-Alg@CTs hydrogel beads external surface indicating a high mass transfer rate. The second phase involves intraparticle diffusion, where metal ions penetrate the interior of Na-Alg@CTs hydrogel beads and adsorb into the internal pore surface. The final phase represents the equilibrium state of adsorption. The diffusion rate constants K_IPD_ were ranked as K_IPD, 1_ > K_IPD, 2_ > K_IPD, 3_ corresponding to external surface adsorption, intraparticle diffusion to the internal surface, and equilibrium stage, respectively [54].

#### 3.4.5. Thermodynamic Study

A thermodynamic study was conducted to obtain detailed information on the inherent energetic changes associated with the adsorption of Cu (II) and Zn (II). The thermodynamic parameters including Gibbs free energy change (∆G°), enthalpy change (∆H°, kJ/mol), and entropy change (∆S°, J/mol.k) were calculated using van’t Hoff’s law, which is described in the following equations [55].

(8) $$\Delta G{}^{\circ}=-RT\;ln\;\left(K_{D}\right)$$

(9) $$Ln\;\left(K_{D}\right)=\frac{\Delta S}{R}-\frac{\Delta H}{RT}$$

(10) $$K_{D}=\frac{Q_{m}}{C_{e}}1000$$

where T (K), K_D_ (L/mol), and R (8.314 J·mol·K^−1^) are the temperature, distribution coefficient, and constant of gas.

In this work, Zn (II) and Cu (II) adsorption by Na-Alg@CTs hydrogel beads was evaluated at three different temperatures (298, 308, and 318 K); Figure S3 shows van’t Hoff’s law. Based on Table 3, The negative values of ΔG° indicate that Zn (II) and Cu (II) were spontaneously adsorbed onto the surface sites of Na-Alg@CTs hydrogel beads [56]. Furthermore, the decreasing absolute values of ΔG° with increasing temperature indicate that higher temperatures enhance adsorption. The positive ΔH° value proves that the heavy metals adsorption is endothermic, while the positive ΔS° value demonstrates an increase in disorder at the interface between the adsorbent and the adsorbate during the adsorption process [57].

## 4. Conclusions

In summary, Na-Alg@CTs hydrogel beads were synthesized using *Cellana Tramoscrica* seashells as a natural source of calcium carbonate, which was encapsulated within a sodium alginate matrix. XRD and FTIR analysis techniques confirmed the successful synthesis, evidenced by reduced band intensities. The hydrogel beads were applied as adsorbents for Cu (II) and Zn (II), exhibiting excellent adsorption properties, as demonstrated by SEM and EDS analysis. Adsorption behavior was well described by the Langmuir isotherm model, yielding maximum adsorption capacities (Qm) of 368.85 mg/g for Cu (II) and 1075.67 mg/g for Zn (II). The kinetic analysis indicated that the adsorption adhered to the pseudo-second-order (PSO) model. Additionally, the thermodynamic experiment showed that the process was spontaneous and endothermic, suggesting favorable adsorption at higher temperatures. These results underscore the potential of seashell-derived Na-Alg@CTs hydrogel beads as a low-cost and environmentally friendly adsorbent for removing Cu (II) and Zn (II) from wastewater.

## Figures and Tables

**Table 1:** Isotherm parameters for Cu (II) and Zn (II) adsorption by Na-Alg@CTs hydrogel beads.

Isotherm	Parameters	Cu (II)	Zn (II)
	Q_e,exp_ (mg/g)	342.09	1032.371
**Langmuir**	Q_m_ (mg/g)	368.857	1075.676
	K_L_ (L/mg)	0.106	0.075
	χ^2^	59.97	813.249
	RMSE	7.744	28.517
	R^2^	0.996	0.979
**Freundlich**	K_F_	77.133	203.538
	n_F_	3.00	3.199
	χ^2^	644.38	8238.431
	RMSE	25.384	90.76
	R^2^	0.964	0.953

**Table 2:** Kinetic data of Cu (II) and Zn (II) adsorption using Na-Alg@CTs hydrogel beads.

Model	Parameters	Cu^2+^	Zn^2+^
	Qe, exp (mg/g)	18.23	17.97
**PFO**	K_1_ (min^−1^)	0.114	0.279
	Q_1_ (mg/g)	16.643	17.057
	R^2^	0.866	0.953
	χ^2^	3.781	1.17
**PSO**	K_2_ (g mg^−1^ min^−1^)	0.009	0.030
	Q_2_ (mg/g)	17.770	17.648
	R^2^	0.949	0.983
	χ^2^	1.648	0.40
**IPD**			
**First phase**	K_IPD,1_	1.002	0.753
	C	8.206	12.026
	R^2^	0.889	0.964
**Second phase**	K_IPD,2_	0.725	0.438
	C	9.025	13.054
	R^2^	0.996	0.989
**Third phase**	K_IPD,3_	0.439	0.134
	C	12.391	16.145
	R^2^	0.583	0.756

**Table 3:** Thermodynamic parameters of Cu (II) and Zn (II) onto Na-Alg@CTs hydrogel beads.

Adsorbate	∆S (J/mol.k)	∆H (KJ/mol)	∆G (KJ/mol)		
			298 K	308 K	315 K
Cu (II)	140.34	15.33	−26.48	−27.89	−28.87
Zn (II)	138.73	15.53	−25.81	−27.19	−28.16

## Data Availability

The original contributions presented in the study are included in the article/Supplementary Material, further inquiries can be directed to the corresponding authors.

## Supplementary Materials

The following supporting information can be downloaded at: https://www.mdpi.com/article/10.3390/polym16233257/s1, Figure S1: Preparation protocol of Na-Alg@CTs hydrogel beads; Figure S2: EDS spectrum of (a) CTs, (b) Na-Alg@CTs hydrogel beads, (c) Cu-Na-Alg@CTs, and (d) Zn-Na-Alg@CTs; Figure S3: van’t Hoff law for (a) Cu (II) and (b) Zn (II) uptakes on Na-Alg@CTs hydrogel beads.
