# Supplementary material for: Eco-Friendly Hydrogel Beads from Seashell Waste for Efficient Removal of Heavy Metals from Water
Source: Polymers (Basel). 2024 Nov 23;16(23):3257. doi: 10.3390/polym16233257 (PMC11644139; doi:10.3390/polym16233257)
Supplement: Supplementary file 1 [file polymers-16-03257-s001.zip › polymers-3298476-supplementary.pdf]

Eco-Friendly Hydrogel Beads from Seashell Waste for Efficient Removal of Heavy Metals from Water

Zaineb Mchich [1], Daniela Simina Stefan [2,*], Rachid Mamouni [1,*], Nabil Saffaj [1] and Magdalena Bosomoiu [2]

[1] Team of Biotechnology, Materials, and Environment, Faculty of Sciences, Ibn Zohr University, Agadir BP 8106, Morocco; zaineb.mchich@edu.uiz.ac.ma (Z.M.); n.saffaj@uiz.ac.ma (N.S.)
[2] Department of Analytical Chemistry and Environmental Engineering, Faculty of Chemical Engineering and Biotechnologies, National University of Science and Technology Politehnica of Bucharest, 1-7 Polizu Street, 011061 Bucharest, Romania; magdalena.bosomoiu@upb.ro
* Correspondence: daniela.stefan@upb.ro (D.S.S.); r.mamouni@uiz.ac.ma (R.M.)

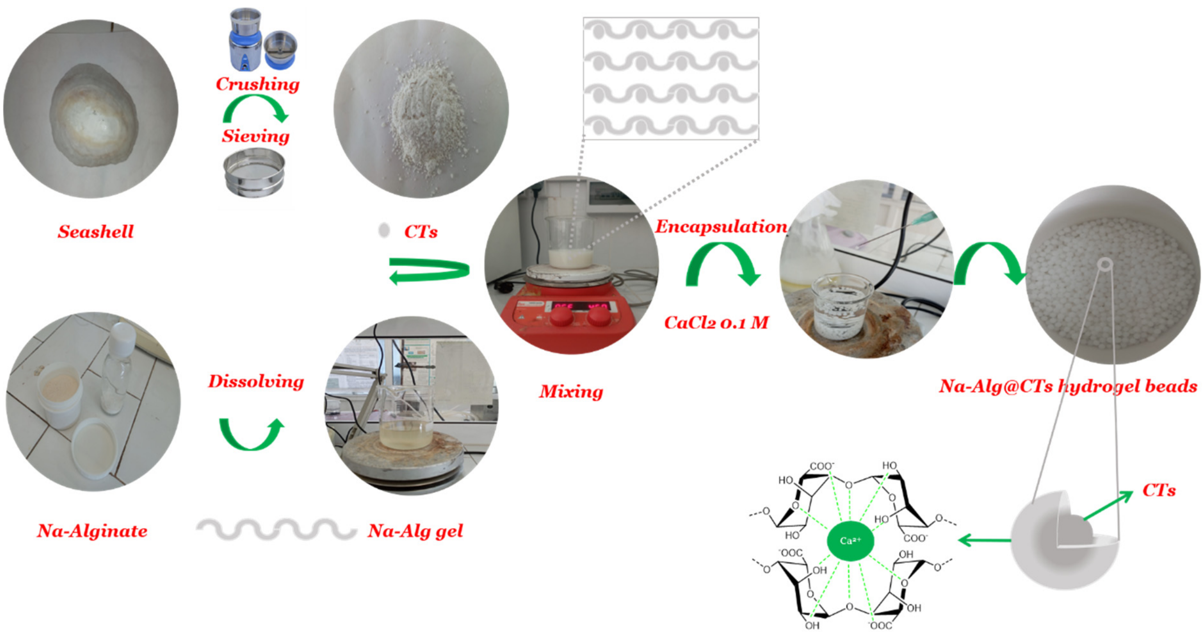

Figure S1: Preparation protocol of Na-Alg@CTs hydrogel beads.

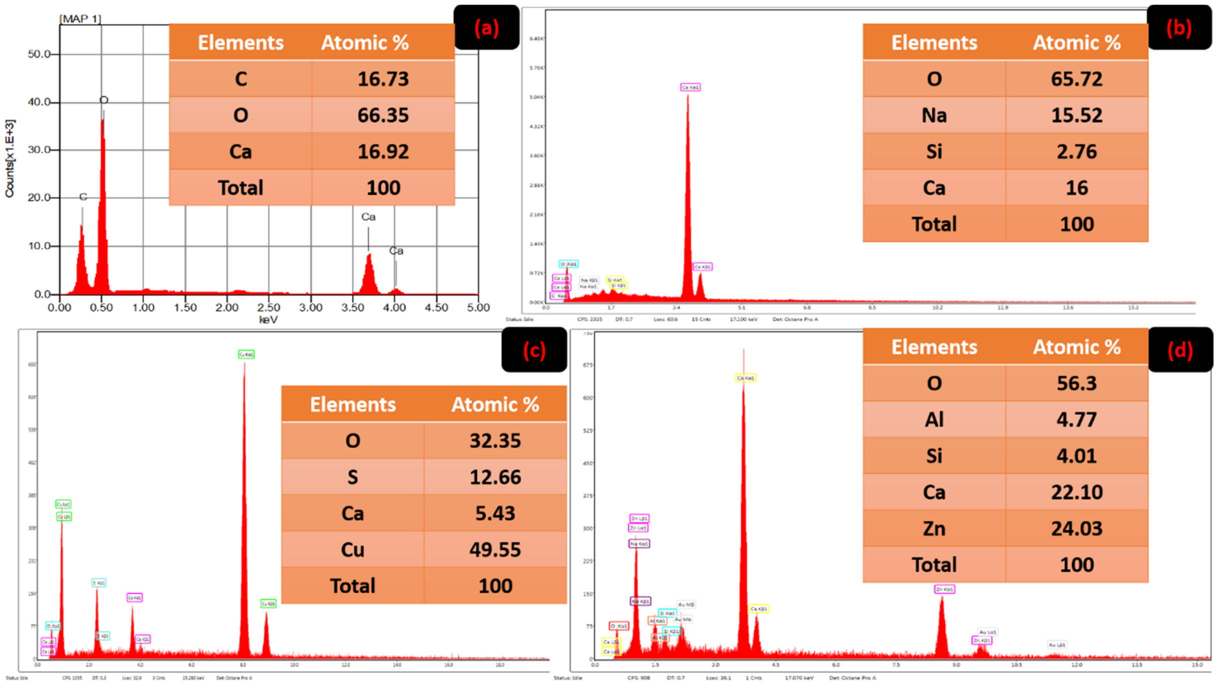

Figure S2: EDS spectrum of (a) CTs, (b) Na-Alg@CTs hydrogel beads, (c) Cu-Na-Alg@CTs, and (d) Zn-Na-Alg@CTs.

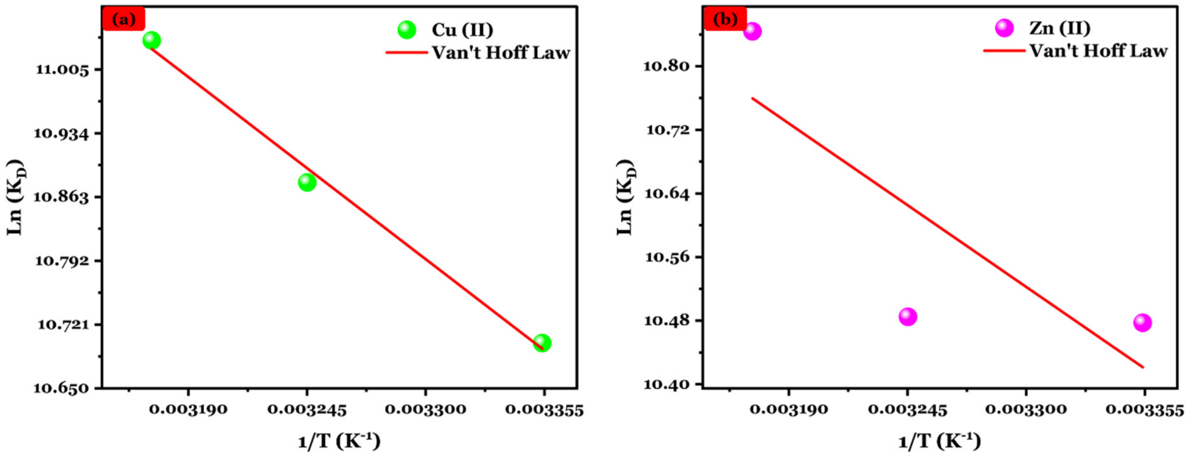

Figure S3: van't Hoff law for (a) Cu (II) and (b) Zn (II) uptakes on Na-Alg@CTs hydrogel beads.
